# Peer Support Specialists’ Perspectives of a Standard Online Research Ethics Training: Qualitative Study

**DOI:** 10.2196/29073

**Published:** 2022-02-01

**Authors:** Karen L Fortuna, Skyla R Marceau, Arya Kadakia, Sarah I Pratt, Joy Varney, Robert Walker, Amanda L Myers, Shavon Thompson, Katina Carter, Kaycie Greene, Willie Pringle

**Affiliations:** 1 Department of Psychiatry Geisel School of Medicine Dartmouth College Lebanon, NH United States; 2 Geisel School of Medicine Dartmouth College Concord, NH United States; 3 Dartmouth College Hanover, NH United States; 4 Centerstone Kentucky Louisville, KY United States; 5 Office of Recovery and Empowerment Massachusetts Department of Mental Health Boston, MA United States; 6 Institute for Behavioral Health Heller School Brandeis University Waltham, MA United States; 7 Centerstone Tennessee Clarksville, TN United States

**Keywords:** peer support specialists, community engagement, research ethics, mental health, peer support, codebook, online health, online training, education, ethics

## Abstract

**Background:**

Certified peer support specialists (CPS) have a mental health condition and are trained and certified by their respective state to offer Medicaid reimbursable peer support services. CPS are increasingly involved as partners in research studies. However, most research ethics training in the protection of human subjects is designed for people who, unlike CPS, have had exposure to prior formal research training.

**Objective:**

The aim of this study is to explore the perspectives of CPS in completing the Collaborative Institutional Training Initiative Social and Behavioral Responsible Conduct of Research online training.

**Methods:**

A total of 5 CPS were recruited using a convenience sample framework through the parent study, a patient-centered outcomes research study that examined the comparative effectiveness of two chronic health disease management programs for people with serious mental illness. Participants independently completed the Collaborative Institutional Training Initiative Social and Behavioral Responsible Conduct of Research online training. All participants completed 15 online modules in approximately 7-9 hours and also filled out a self-report measure of executive functioning (the Adult Executive Functioning Inventory [ADEXI]). Qualitative data were collected from a 1-hour focus group and qualitative analysis was informed by the grounded theory approach. The codebook consisted of codes inductively derived from the data. Codes were independently assigned to text, grouped, and checked for themes. Thematic analysis was used to organize themes.

**Results:**

Passing scores for each module ranged from 81%-89%, with an average of 85.4% and a median of 86%. The two themes that emerged from the focus group were the following: comprehension (barrier) and opportunity (facilitator). Participants had a mean score of 27.4 on the ADEXI.

**Conclusions:**

The CPS perceived the research ethics online training as an opportunity to share their lived experience expertise to enhance current research efforts by nonpeer scientists. Although the CPS completed the online research ethics training, the findings indicate CPS experienced difficulty with comprehension of the research ethics online training materials. Adaptations may be needed to facilitate uptake of research ethics online training by CPS and create a workforce of CPS to offer their lived experience expertise alongside peer and nonpeer researchers.

## Introduction

It is widely recognized that the inclusion of the insights of people with mental health conditions in psychiatric research is needed to advance mental health care [1]. Certified peer support specialists (CPS) are people with a lived experience of a mental health condition who have been trained and accredited by their state to provide Medicaid reimbursable support services [2], including peer support [3]. Peer support is a nonmanualized, nonstandardized form of social support that a person provides to others experiencing a mental health and/or chronic health condition to bring about change [2]. Peer support has been shown to increase individuals’ hope, their sense of personal control, and their ability to make positive changes, and decrease psychiatric symptoms [4] through listening, sharing one’s lived experiences, and role modeling [2]. The inclusion of CPS into the workforce has transformed the mental health system globally in that peer support services have the capacity to support people in between clinical encounters. As such, CPS is one of the fastest-growing sectors of the mental health workforce providing community-based services [5].

CPS are also increasingly involved in research-related activities (eg, recruitment, retention efforts, obtaining informed consent, collecting sensitive data, publishing findings in peer-reviewed journals [6]). For example, the Quality of Patient-Centered Outcomes Research Partnerships instrument was developed by researchers and CPS to offer quality improvement opportunities related to developing equitable research partnerships [7]. Moreover, community-engaged research that includes CPS in all stages of research may also lead to the dissemination of study results to a wider audience by incorporating CPS in conferences and publications as well as by using social media to showcase results in a more digestible manner for service users of the mental health system and other CPS [6].

As the influence of CPS in research grows, adequately training CPS in research ethics human subjects protection is an emerging concern [8]. Since regulations that required organizations to establish institutional review boards have been instituted, multiple online training programs in research ethics have been developed, such as the Collaborative Institutional Training Initiative (CITI). The CITI Program covers various domains, such as human subject research, and includes courses on various topics (eg, research ethics, protocol development, and safety practices). Each course includes webinars, text, and quizzes that overall take approximately 30-45 minutes to complete [9]. Such courses are designed for university researchers and students, as well as health care providers and governmental agencies, for basic accreditation [10]. Human research ethics training currently exists for special populations, including Aboriginal peoples [11], faith-based communities [12], community health workers [13], and *Promotores* (ie, Latino community mental health workers) [13]. Nevertheless, while these trainings target lay populations with limited research backgrounds, no human research ethics trainings (to our knowledge) have been designed specifically to address the cognitive challenges these trainings pose to individuals new to research who may also experience a mental health condition.

Hence, the purpose of this study was to examine the perspectives of CPS in completing the CITI Social and Behavioral Responsible Conduct of Research online training. Findings can be used to determine the feasibility of current community-engaged research training and identify opportunities for improvement.

## Methods

### Overview

A total of 5 CPS were hired as part of a Patient-Centered Outcomes Research Institute study. The parent study is in the process of conducting a randomized control trial (N=600 people with a lived experience of a mental health condition) to examine the impact of two chronic health disease management programs for people with serious mental illness. The outcomes of interest include change in knowledge and skills related to illness self-management, patient activation, and acute hospital events. As part of this study, researchers employed the Peer and Academic Partnership model, which has been described in detail elsewhere [6]. Briefly, CPS were hired and financially reimbursed to work as partners in the implementation of the randomized control trial detailed above. To be eligible, individuals had to meet the following criteria: (1) have lived experience of any mental health condition, (2) have a peer support certification, (3) reside in the United States, and (4) be aged ≥18 years. Prior to conducting that study, the CPS did not have prior research training or research experience. As part of the parent study, the CPS met monthly through videoconference with a trained PhD researcher (first author) to provide input on randomized control trial implementation challenges (eg, recruitment, retention). Improvements included inclusion of innovative recruitment strategies and development of new COVID-19–related intervention materials. For this study, a convenience sample framework of those CPS hired to provide patient-centered insights for the implementation of the parent study was used. The participants were hired to be part of this study if they had time available and had an interest in taking part in the research. All participants were already employees of the organization. Prior to completing the online research ethics training, the voluntary and confidential nature of this study was explained, and verbal informed consent was obtained. This study was approved by the Dartmouth College Institutional Review Board.

Participants then completed the CITI Social and Behavioral Responsible Conduct of Research online training that consisted of 15 online modules: (1) Belmont Report and Its Principles, (2) Conflicts of Interest in Human Subjects Research, (3) Cultural Competence in Research, (4) History and Ethical Principles, (5) Defining Research with Human Subjects, (6) The Federal Regulations, (7) Assessing Risk, (8) Informed Consent, (9) Course Privacy and Confidentiality, (10) Research with Prisoners, (11) Research with Children, (12) Research in Public Elementary and Secondary Schools, (13) International Research, (14) Course Internet-Based Research, and (15) Unanticipated Problems and Reporting Requirements in Social and Behavioral Research. Participants completed the modules online independently and had their scores emailed to the first author (KLF) along with their passing certificate. The first author was present to help while they completed the training in a single session. They were financially reimbursed regardless of whether they passed the training or not.

Following the completion of the online research ethics training, a 1-hour focus group was cofacilitated by a trained PhD-level facilitator (KLF) and a masters-level CPS (JV) to collect qualitative data. An interview guide aided the cofacilitators (see Textbox 1). The PhD-level facilitator and the masters-level CPS, both of whom have extensive research experience and training, developed the interview guide. The participants were encouraged to express their views of the online research ethics training and their role as a CPS research partner. No follow-up interviews were conducted. The focus group was audio recorded and the data were transcribed. The focus group was conducted in October 2019 and lasted approximately 1 hour.

**Focus group interview guide.**

**Perspectives on the Social and Behavioral Responsible Conduct of Research training**

1. What are your views on clinical research in general?

2. What are your thoughts on the content of the Social and Behavioral Responsible Conduct of Research training?

3. What are your thoughts on the quizzes in the Social and Behavioral Responsible Conduct of Research training?

4. How would you feel as a peer support specialist being part of a research team?

5. How did you feel going through the training?

### Study Sample

The sample included 5 CPS from 2 states. The majority of participants were female (n=4, 80%) and African American (n=4, 80%). Ages ranged from 40-52 years, with a mean age of 45.8 years. All participants had a high school education/General Educational Development certificate and were providing services in their respective communities. Tenure in CPS positions ranged from 3 months to 4 years. None of the CPS had completed research ethics training in the past or had any research experience. As CPS are protected by the Americans with Disabilities Act, data on their diagnosis remained confidential.

### Instruments

Each participant also completed the Adult Executive Functioning Inventory (ADEXI), a self-report measure that assessed their executive functioning. It is a reliable 14-item self-report scale (Table 1) assessing working memory and inhibition in adulthood using a 5-point Likert scale ranging from 1 (definitely not true) to 5 (definitely true). The summed score (ranging from 14-70) is used to assess executive functioning. Higher scores indicate greater difficulty with executive functioning.

**Table 1:** Adult Executive Functioning Inventory.

Number	Statement^a^
1	I have difficulty remembering lengthy instructions.
2	I sometimes have difficulty remembering what I am doing in the middle of an activity.
3	I have a tendency to do things without first thinking about what could happen.
4	I sometimes have difficulty stopping myself from doing something that I like even though someone tells me that it is not allowed.
5	When someone asks me to do several things, I sometimes remember only the first or last.
6	I sometimes have difficulty refraining from smiling or laughing in situations where it is inappropriate.
7	I have difficulty coming up with a different way of solving a problem when I get stuck.
8	When someone asks me to fetch something, I sometimes forget what I am supposed to fetch.
9	I have difficulty planning for an activity (eg, remembering to bring everything necessary when going on a trip/to work/to school).
10	I sometimes have difficulty stopping an activity that I like (eg, I watch TV or sit in front of the computer in the evening even though it is time to go to bed).
11	I sometimes have difficulty understanding verbal instructions unless I am also shown how to do something.
12	I have difficulties with tasks or activities that involve several steps.
13	I have difficulty thinking ahead or learning from experience.
14	People that I meet sometimes seem to think that I am more lively/wilder compared to other people my age.

^a^Participants are asked to circle a number to indicate how well that statement describes how they are as a person (1=definitely not true, 2=not true, 3=partially true, 4=true, and 5=definitely true).

### Analytic Plan

Qualitative analysis was informed by the “grounded theory” approach [14]. The codebook included codes inductively derived from qualitative data [14]. KLF and SRM read transcribed qualitative data. Codes were independently assigned to text, grouped, and thematically analyzed to check for emerging themes. Analyses included within-group consensus or disagreement. Member checking with CPS was used to verify and/or resolve any dissimilar findings. Member checking is a method used to validate findings that involves discussing findings with respondents and examining the accuracy of findings [15].

## Results

### Social and Behavioral Responsible Conduct of Research Training

A total of 5 CPS independently completed the CITI Social and Behavioral Responsible Conduct of Research online training. All participants completed 15 online modules. Completion of the research ethics training took approximately 7-9 hours. Scores were calculated and collected through the CITI platform and were then emailed to KLF. Passing scores for each module ranged from 81%-89%, with an average of 85.4% and a median of 86%.

We identified a final set of 9 codes (ie, sentence structure, grade level, length, new knowledge, acronyms, cognitive load and emotional response retention, retention recommendations, lived experience expertise, and new opportunity) relating to 2 themes from the focus group. Themes related to peer support specialists’ perspective of the barriers and facilitators to completing the research ethics training included the following: comprehension (barrier) and opportunity (facilitator).

### Comprehension

The first theme, comprehension, included two subcategories: (1) cognitive complexity of content and quizzes and (2) learning and retention.

#### Cognitive Complexity of Content and Quizzes

All CPS respondents referred to the cognitive complexity of the content and quizzes as a barrier to completing the research ethics training (eg, “give me simple sentences. That was too much”). Sentence structure was multisyllabic and written at a 12th grade level (as assessed by the Flesch-Kincaid Grade Level test in Microsoft Word), which required extensive cognitive effort (eg, “The big words, the paragraph questions, I like that it was only five questions but they were just long, they were real big”).

#### Learning and Retention

All CPS respondents referred to learning and retention as a barrier to completing the research ethics training. Most CPS respondents (n=4, 80%) indicated the need to comprehend information to retain knowledge (eg, “I couldn't even say oh that's interesting because my brain was so overwhelmed by the way the content was written?”). Unfamiliarity with terms and acronyms such as “NIH” or “PHS” led to confusion, and produced feelings of frustration and exhaustion in completing the training. CPS respondents recommended hyperlinks for unfamiliar terms to assist CPS test takers in understanding definitions of words.

### Opportunity

All CPS respondents perceived research ethics education as an opportunity to share their lived experience expertise to enhance current research efforts by nonpeer scientists (eg, “If the world is open to it I think peer support specialists could have a great role in research because we have a different perspective... we come from a perspective of experience” and “this is a once-in-a-lifetime opportunity”).

### ADEXI

Participants had a mean executive functioning score of 27.4 (SD 7.83) on the ADEXI. Participants had a mean working memory score of 17.2 (SD 5.50) and inhibition score of 10.2 (SD 2.59).

## Discussion

### Principal Findings

The purpose of this study was to explore the perspectives of CPS regarding an online research ethics training. All participants completed the CITI Social and Behavioral Responsible Conduct of Research online training. The findings indicate difficulty with comprehension of research ethics online training materials. The ADEXI scale indicated that participants had little self-reported difficulty with executive functioning. This suggests that perhaps CPS involved in this study were not hindered by poor working memory and inhibition, and that difficulties emerged from research-specific complexity such as the use of jargon and the need to absorb new and unfamiliar information. Nevertheless, CPS perceived the research ethics online training as an opportunity to share their lived experience expertise to enhance current research efforts by nonpeer scientists.

All participants completed the CITI research ethics training in 7-9 hours—approximately 2.5-4.5 hours longer than the average completion time [16]. As CPS are increasingly involved in research as partners, adaptations may be needed to facilitate the uptake of online research ethics training. Adaptations can include providing information on the time commitment needed to complete these tests, multiple testing sessions on different days, technological capacity to sign in and out of training portals and begin where one had left off, and opportunities to complete testing at home.

The cognitive complexity of content and quizzes can be addressed by reducing the number of multisyllabic words and sentences that included compound subjects, which required extensive cognitive effort to comprehend. Although programs that provide research ethics training specifically for community research partners exist—for example, CIRTification: Community Involvement in Research Training—these community research partner trainings are designed for people with a high school education [17]. Although such trainings may be more digestible for CPS (as compared to the CITI training, which requires prior research experience), many CPS may not meet the educational requirement, as a high school education is not required to become a CPS in many states [18]. Existing research suggests that plain-language summaries and the use of language written at a fourth-grade level [19] could improve the readability and comprehensibility of trainings—thus, existing research ethics trainings could be improved by following plain-language summary and reading-level recommendations.

The use of acronyms such as “NIH” or “PHS” in research ethics training impacted comprehension. Making meanings explicit is an essential web-based design feature necessary to facilitate learning among people with mental health conditions [20]. Including definitions can improve comprehension by making meanings explicit. CPS respondents recommended hyperlinks in web-based applications to link test takers to definitions of words [20]. Evidence-based guidelines for this population support this finding [20]. Additional evidence-based, web-based design guidelines that may be useful in online research ethics training include a singular focus in website content, simple architecture, prominent content, and explicit navigation [20].

As they did not have prior knowledge of research processes, terminology, and the history of research ethics, CPS were learning new information. Determining the factors that influence how CPS learn may aid researchers in understanding better ways to help CPS grasp new content. Research indicates that factors that may impact the learning experience of CPS include the following: (1) life experiences, (2) work experience, (3) previous adult learning experiences [21,22], and (4) potential cognitive deficits related to mental health conditions [23]. As such, real-world examples in the context of research ethics training may facilitate learning and retention of new knowledge. In addition, people with mental health conditions may have potential cognitive impairments and need repetition to reinforce new knowledge [20]. Exploring ongoing learning methods such as “audit and feedback” or “learning collaboratives” may offer opportunities to facilitate reinforcement of research ethics training and lead to greater retention of new knowledge [20].

All CPS respondents perceived research ethics education as an opportunity to share their lived experience expertise to enhance current research efforts by nonpeer scientists.

This study is not without limitations. First, this study included a nonprobability convenience sample. As such, the study sample may not be representative of the greater population of CPS. In addition, the severity of mental health conditions among CPS is not known. Although the severity of a mental health condition could impact comprehension of CITI modules, CPS are employees of organizations, and therefore the Americans with Disabilities Act protects them from reporting on a diagnosis they have been given. As such, we did not request information related to a participant’s mental health diagnosis. Second, due to the small sample size, it is not known if saturation was met. However, this study is the first to explore peer support specialists’ perceptions of human research ethics training and delineates recommendations to develop an online training for this group. Third, it is not known if participants needed to take a CITI module more than once before receiving a passing score. Consequently, it would have been informative to collect the number of times a participant needed to retake a module to pass. In addition, our sample was largely from the same demographic, and it is possible that stratifying by different demographics may produce alternate outcomes. Finally, we examined only one standard online research ethics training. Other trainings for community partners may be better suited for CPS. However, to our knowledge, standard community research partner trainings are designed for people with a high school education [17]. A research ethics training designed for community health workers [13] may potentially have higher levels of acceptability among CPS. Nevertheless, this is the first study, to our knowledge, to examine perspectives of CPS in completing an online research ethics training.

Next steps could potentially include the development of an ethics training that is tailored to research-naive individuals and those with limited education using universal design principles. Universal design is the process of creating products that are accessible to people with a wide range of abilities [24]. Based on our findings, we suggest reducing verbosity and the use of jargon. A more fundamental explanation of the importance of research ethics and the inclusion of more definitions for certain terms would reduce the cognitive complexity of such training for many CPS. Concurrently, repetition—along with the use of visual aids and demo videos—would aid in the learning and retention of the content. Moreover, ease of navigation can play a critical role in how peer specialists interact with the content they need to learn [25]. Improving usability would consequently help CPS maximize opportunities to share their lived experience with researchers. Future studies should use a larger sample size and include CPS from multiple demographics to better examine how CPS respond to research ethics trainings.

### Conclusion

As the inclusion of CPS as research partners with shared decision-making authority becomes more commonplace, this may result in complications in research if research ethics training is not designed for this population. Although research ethics training programs for community research partners exist, they may need further adaptation for CPS.

## Abbreviations

ADEXI: Adult Executive Functioning Inventory
CITI: Collaborative Institutional Training Initiative
CPS: certified peer support specialist
